# Depression, Anxiety and Stress among Nurses Providing Care to the COVID-19 Patients: A Descriptive Cross-sectional Study

**DOI:** 10.31729/jnma.7235

**Published:** 2022-02-28

**Authors:** Menuka Bhandari, Upendra Yadav, Tulasha Dahal, Anjula Karki

**Affiliations:** 1 Biratnagar Nursing Campus, Institute of Medicine, Tribhuvan University, Biratnagar, Morang, Nepal; 2 B.P. Koirala Institute of Health Science, Dharan, Sunsari, Nepal; 3 Koshi Hospital, Biratnagar, Morang, Nepal

**Keywords:** *anxiety*, *COVID-19*, *depression*, *nurses*, *stress disorder*

## Abstract

**Introduction:**

Addressing adequately the mental health during and after COVID-19, as well as preparation for possible future outbreaks, requires an understanding of the nature and extent of mental health impacts, factors related to negative mental health outcomes and symptoms of mental illness. The aim of this study was to find the prevalence of depression, anxiety and stress among nurses providing care to the COVID-19 patients.

**Methods:**

A descriptive cross-sectional study was conducted from 10^th^ April 2021 to 30^th^ June 2021 among 301 nurses from three COVID-19 dedicated hospitals using self-administered questionnaires. Whole sampling technique was used. Ethical approval was obtained from the Ethical Review Board of Nepal Health Research Council (Registration number: 106/2021P). The data was analyzed using the Statistical Package for the Social Sciences version 16. Descriptive statistics like frequency, percentage, mean and standard deviation were calculated.

**Results:**

Out of 301 nurses, the prevalence of depression, anxiety and stress was 258 (85.72%), 189 (62.80%) and 151 (49.84%) respectively.

**Conclusions:**

The study has shown a higher prevalence of depression, anxiety and stress among nurses in comparison to other studies in the similar settings. A quick assessment of the mental health status and mental health requirements of nurses would be helpful in responding to and reducing psychological distress in the crisis situation. The mental health status of nurses should thus be closely monitored by the employing health institutions including managing their workload, providing emotional support and responding to their personal needs.

## INTRODUCTION

SARS Coronavirus 2 (SARS-Cov-2) triggered the Coronavirus Disease 2019 (COVID-19) outbreak which was declared as a global pandemic by the World Health Organization (WHO) on March 11, 2020. The first case in Nepal was detected on January 23, 2020 and the number of cases increased rapidly.^1,2^ Nurses are more vulnerable to sadness, anxiety and stress due to the demanding nature of their work which could impair performance and self-efficacy.^3,4^

Nurses worried about infecting their family, friends and coworkers, felt insecure and stigmatized ,and reported high levels of stress, anxiety and depressive symptoms with reluctance to work, which could have long-term psychological implications.^5,6^ Various problems like inadequate provision of Personal Protective Equipment (PPE), incentives, government facilities and mass resignation by nurses in COVID-19 dedicated hospitals were the cause of interest for this study.

The main objective of this study was to find out the prevalence of depression, anxiety and stress among nurses in COVID-19 hospitals.

## METHODS

This was a descriptive cross-sectional study conducted among nurses involved in the care of COVID-19 patients in 3 COVID hospitals of Province 1, namely Koshi COVID Treatment Center, Biratnagar; B.P. Koirala Institute of Health Sciences (BPKIHS), Dharan; and Nobel Medical College Teaching Hospital, Biratnagar from April 10 to June 30, 2021. There were total 130 nurses in Koshi hospital, around 500 nurses in BPKIHS Dharan and 350 in Nobel Medical College Teaching Hospitals. Among them, 301 nurses were assigned to the care of COVID-19 patients during that period. Ethical clearance was obtained from Ethical Review Board of Nepal Health Research Council (NHRC) (Registration number: 106/2021P). Whole sampling technique was done.

The self-administered questionnaire was used which consisted of socio-demographic variables, degree of concern towards COVID-19 care variables, and Nepali Version of DASS-21. The 5 point Likert scale was used to measure the depression, anxiety, and stress of nurses. Tonsing K, et al. assessed the psychometric properties and validation of the Nepali version of the Depression Anxiety Stress Scales (DASS-21) and demonstrated adequate psychometric properties in relation to internal consistency and validity.^7^

The data was then analyzed using the Statistical Package for the Social Sciences (SPSS) version 16. Descriptive statistics like frequency, percentage, mean and standard deviation were calculated.

## RESULTS

Out of 301 nurses, the prevalence of depression, anxiety and stress was 258 (85.72%), 189 (62.80%), and 151 (49.84%) respectively. Among them, 104 (34%) nurses have seen mild depression, 13 (4.31%) have moderate depression, 13 (4.31%) have severe depression and only three (0.99%) have extreme depression according to their response. Similarly, 27 (8.97%) nurses have mild anxiety, 130 (43.18%) have moderate anxiety, 101 (33.55%) have severe anxiety, and 32 (10.63%) have extreme anxiety and regarding stress, 107 (35.54%) nurses have mild stress, 37 (12.29%) have moderate stress, and six (1.99%) have severe stress (Table 1).

**Table 1:** Prevalence of depression, anxiety and stress symptoms among respondents (n= 301).

Variables	Mild n (%)	Moderate n (%)	Severe n (%)	Extreme n (%)
Depression	104 (34.55)	138 (45.84)	13 (4.31)	3 (0.99)
Anxiety	27 (8.97)	130 (43.18)	101 (33.55)	32 (10.63)
Stress	107 (35.54)	37 (12.29)	6 (1.99)	-

The mean age was 26.72±6.28 years, the majority of nurses i.e. 175 (58.1%) belonged to less than 25 years. More than half 205 (68.1%) completed Proficiency Certificate Level (PCL) nursing and the majority of the nurses 241 (80.1%) were staff nurses. Total of 196 (65.1%) had less than five-year experience, 179 (60%) were unmarried and 122 (40%) were married. The proportion of nurses represented were 130 (43.2%) from Nobel Medical College, 100 (33.2%) from BPKIHS Dharan, and 71 (23.6%) from Koshi COVID treatment center respectively (Table 2).

**Table 2:** Sociodemographic characteristics of nurses in the study (n= 301).

Variables	Categories	n (%)
Age (years)	<25	175 (58.1)
	26-35	103 (34.2)
	36-45	18 (6)
	46 and above	5(1.7)
Education	Auxiliary Nurse Midwife (ANM)	11 (3.7)
	PCL	205 (68.1)
	Bachelor	78 (25.9)
	Master and above	7 (2.3)
Position	ANM	16 (5.3)
	Staff nurse	241 (80.1)
	Nursing officer	39 (13)
	Nursing administrator	5(1.7)
Experience (years)	<5	196 (65.1)
	6-10	61 (20.3)
	11-15	22 (7.3)
	16 and above	22 (7.3)
Marital Status	Married	122 (40.5)
	Unmarried	179 (59.5)
Hospitals	Nobel Medical College	130 (43.2)
	BPKIHS Dharan	100 (33.2)
	Koshi Hospital	71 (23.6)

While measuring the level of concern to COVID-19 related variables, more than half 199 (66.1%) nurses were extremely concerned about personal protective equipment (PPE), 186 (61.8%) were extremely concerned about staff quarantine, 174 (57.7%) were extremely concerned about COVID care, 165 (54.8%) personnel safety, 143 (47.5%) family safety, 136 (45.2%) adequate human resources, 125 (41.5%) adequate kits, 117 (38.9%) motivation package and 118 (39.2%) staffing (Table 3).

**Table 3:** Respondents concerned to COVID-19 related variables (n= 301).

Variables	Not concerned at all (F/P)	Not very concerned (F/P)	Somewhat concerned (F/P)	Moderately concerned (F/P)	Extremely concerned (F/P)
COVID care	8 (2.7)	4 (1.3)	25 (8.3)	90 (29.9)	174 (57.8)
PPE	3 (1)	6 (2)	17 (5.6)	76 (25.2)	199 (66.1)
Adequate kits	7 (2.3)	23 (7.6)	56 (18.6)	90 (29.9)	125 (41.5)
Training	31 (10.3)	23 (7.6)	40 (13.3)	92 (30.6)	115 (38.2)
Staffing	8 (2.7)	9 (3)	50 (16.6)	116 (38.5)	118 (39.2)
Personal safety	5(1.7)	5(1.7)	35 (11.6)	91 (30.2)	165 (54.8)
Family safety	26 (8.6)	16 (5.3)	24 (8)	92 (30.6)	143 (47.5)
Human resources	15 (5)	16 (5.3)	37 (12.3)	97 (32.2)	136 (45.2)
Motivation package	23 (7.6)	27 (9)	38 (12.6)	96 (31.9)	117 (38.9)
Staff quarantine	13 (4.3)	24 (8)	18 (6)	60 (19.9)	186 (61.8)

## DISCUSSION

Out of 301 nurses, the prevalence of depression, anxiety, and stress was 85.72%, 62.80%, and 49.84% respectively. All the nurses were frontlines and assigned to the care of COVID-19 patients, similar study conducted in Devdaha Medical College revealed that the prevalence of anxiety was 41.4%, depression was 24.1%, and 74% perceived moderate to severe stress.^8^ The current study depicted higher level of depression, anxiety, and stress among nurses than other national studies, this may be due to high social stigma, insecurity in job, forced leave, minimum or lack of motivating factors e.g. inadequate provision of PPE, incentives, isolation facilities, inadequate rest, increased workload, fear of death and isolation from family member and children. Similar findings were displayed by another study i.e. approximately 35% of the participants had extremely severe depression, over 40% had moderate to severe depression, approximately 20% had normal to mild depression, and approximately 60% of the participants, reported extremely severe anxiety.^9^ The findings were also comparable with healthcare workers in Jordan and China.^10,11^

The present study revealed that more than half of nurses were extremely concerned with COVID-19 related facilities; PPE, staff quarantine, and personal safety than other facilities. Access to personal protective equipment for health professionals is also a major worry as the pandemic spreads. Some medical personnel were waiting for equipment while treating patients who may have been infected or were given equipment that did not satisfy standards. A similar study conducted in Wuhan China and Italy indicated that fear of infection, difficulty in controlling the pandemic, and a shortage of medical facilities across the country are all potential reasons for near-high anxiety.^12,13^

Understanding the nature and extent of mental health impacts, factors related to negative mental health outcomes related to psychological distress, and symptoms of mental illness are required to adequately address mental health needs during and after COVID-19, as well as to prepare for possible future outbreaks.^14,15^ Due to a lack of personal protective equipment (PPE), a dangerous work environment, and bad working conditions, employees may see themselves as being at greater risk, as well as worry of transmission to their families. This may result in a lack of drive as well as unpleasant emotions like despair and guilt.^16^ Another finding of the study was that younger health professionals who were aware of government incentives for health workers during COVID-19 were much less likely to be depressed than older and non-knowing health workers.^17^ This indicates that the appropriate stress-coping methods are required to safeguard nurses against psychiatric illness. Research and prevention in mental health may also need to address the unique mental health requirements of healthcare practitioners.^18^

Our research has certain limitations. We were unable to determine psychological distress caused by demographic and other associated factors. The questionnaire had to be self-administered due to strict hospital infection control measures aimed at minimizing contact between healthcare personnel, and the information supplied on symptoms was not checked by a medical practitioner. The findings of the study might be generalizable in only Province 1.

## CONCLUSIONS

This study reported a higher prevalence of depression, anxiety and stress among nurses providing care to the COVID-19 patients as compared to other studies in similar settings. Nurses had a higher proportion of symptoms related to severe anxiety, severe depression and severe stress than other professions. Health workers in Nepal, mostly nurses, are currently working under extreme pressure with limited health resources such as inadequate staffing. The mental health status of nurses should thus be closely monitored by the employing health institutions including managing Conflict of Interest: None. their workload, providing emotional support, and responding to their personal needs.
